# Psychometric properties of the Fear of COVID-19 Scale amongst black South African university students

**DOI:** 10.4102/ajopa.v3i0.57

**Published:** 2021-07-23

**Authors:** Malose Makhubela, Solomon Mashegoane

**Affiliations:** 1 Department of Psychology, Faculty of Humanities, University of Limpopo, Polokwane, South Africa

**Keywords:** COVID-19-related fear, factor structure, students, ordinary fear, validity

## Abstract

Coronavirus disease 2019 (COVID-19) has spread widely leading to a global public health crisis of a pandemic proportion. Whilst infection rates tend to fluctuate in South Africa, COVID-19 remains a life-threatening disease with the capacity to wreak fear and concern. The present study evaluated the psychometric qualities of the Fear of COVID-19 Scale (FCV-19S) amongst black South African university students (*N* = 433; Female: 58%; Mage = 23.51 [SD = 4.18]). The FCV-19S demonstrated a unidimensional factor structure and acceptable internal consistency (α = 0.87), Omega (ω = 0.88) and the greatest lower bound (GLB = 0.90) reliabilities. In addition, discriminant validity was demonstrated when FCV-19S items loaded separately from ordinary fear. The FCV-19S can be used as a measure of COVID-19-related fear amongst black South African university students.

## Introduction

Coronavirus disease 2019 (COVID-19) has spread widely leading to a global public health crisis. Aside from large-scale deaths, one of its consequences has been mental health problems (Kim, Nyengerai, & Mendenhall, 2020). There are predictions that the pandemic-related mental health situation is likely to worsen (Lin, 2020; Qiu et al., 2020). Nowhere has this been felt as in university contexts where there are reports of heightened general distress and anxiety amongst students because of pandemic-associated changes such as lockdown, interrupted academic programmes and migration to online tuition, rendering student life unpredictable (Cao et al., 2020; Dziech, 2020; Hartocollis, 2020).

Fear, a negative emotional response, is one of the likely and natural mental health outcomes when facing life-threatening events such as COVID-19 (Lin et al., 2020; Ng & Kemp, 2020). The emotional response itself may lead to fear-related behaviour, which eventually determines the progress and overall outcome of a major disease outbreak (Shultz et al., 2016). Indeed, much has been said about the impact of fear on general mental health and quality of life (Ford et al., 2019). We are labouring under the assumption that different types of threats to the organism will trigger unique fear responses (Adolphs, 2013). Thus, the fear of COVID-19 is similar to fears associated with invisible and unique infectious diseases such as Ebola. It is likely to be triggered and exacerbated by the incomplete understanding, uncontrollable nature, ever changing status and recently the discovery of more infectious strains of the virus (Roberts, 2021; WHO, 2020). It is exactly this situation that has led researchers to suspect that conventional models and clinical interventions of general anxiety may not work with the fear associated with COVID-19 (Ahorsu et al., 2020; Perz, Lang, & Harrington, 2020; Rajkumar, 2020).

To this end, Ahorsu et al. (2020) developed a seven-item Fear of COVID-19 scale (FCV-19S) to evaluate anxiety specific to COVID-19, based on the protection motivation theory (Rogers, 1975). The measure has been translated into a number of languages and validated in a number of countries including Bangladesh (Sakib et al., 2020), China (Chi et al., 2021), Ethiopia (Elemo, Satici, & Griffiths, 2020), France (Mailliez, Griffiths, & Carre, 2020), Greece (Tsipropoulou et al., 2020), Israel (Bitan et al., 2020), Italy (Soraci et al., 2020), Japan (Masuyama, Shinkawa, & Kubo, 2020) Malaysia (Pang et al., 2020), Mozambique (Giordani, Giolo, Muhl, Estavela, & Gove, 2021), New Zealand (Winter et al., 2020), Russia (Reznik, Gritsenko, Konstantinov, Khamenka, & Isralowitz, 2020), Saudi Arabia (Alyami, Henning, Krägeloh, & Alyami, 2020), Turkey (Satici, Gocet-Tekin, Deniz, & Satici, 2020) and Vietnam (Nguyen et al., 2020). Most of the studies consistently found that the FCV-19S has a unidimensional factor structure.

A few studies have reported a bi-factor structure (Bitan et al., 2020; Chi et al., 2021; Masuyama et al., 2020; Reznik et al., 2020) in varied contexts such as Israel, China, Japan and Russia, respectively. However, Reznik et al. (2020) and Bitan et al. (2020) have been criticised by researchers on account of incorrect use of factor analytic techniques (see Pakpour, Griffiths, & Lin, 2020a; Pakpour et al., 2020b).

It is not clear if the psychometric properties observed in these cited studies will be found in South Africa given the unique socio-cultural context and the status of the pandemic, particularly with university students (Mahlokwane, 2021; Morapela, 22 May; *Sunday Times*, 2021, 28 April). The sparse investigation of psychological measures across diverse populations is shown to account for measurement problems when these measures are applied on the groups they were not validated for (Ramırez et al., 2005). Indeed, to be suitable for use with various groups of people, health outcome measures should at the minimum show that they measure the same constructs across populations. Besides, university students have previously reported high levels of COVID-19-related fear, and the levels (of fear) are associated with depression and anxiety (Elsharkawy & Abdelaziz, 2020; Zolotov, Reznit, Bender, & Isralowitz, 2020). So, measures of COVID-19-related fear with good psychometric evidence are necessary for counselling services at universities, particularly to aid in the identification of students with high levels of COVID-19-associated fear and the prevention of the development of associated mental health problems.

South Africa has seemingly emerged from a second wave of COVID-19 infections and reported a new and more infectious variant of the virus compared with other countries where the FCV-19S has been studied (Farber, 2021; Fink, 2021). Although the roll-out of vaccines has begun in earnest amongst essential health workers and the elderly, there are still uncertainties in the country whether university students will be vaccinated for them (the vaccines) to have any public health impact in institutions of higher learning (Davis, 2021; Govender, 2021). Whilst the spread of COVID-19 is not, by all accounts, completely out of control, there are indications that South Africa is experiencing a ‘third wave’ of infections (Brandt, 2021; Savides, 2021). Rising infection rates have been reported amongst students in universities (Mahlokwane, 2021; Morapela, May 22). For that reason, it is still necessary to validate the FCV-19S for use in a student population. It will be needed when COVID-19 interventions are continuing. Only one study from the United States of America has to date reported on the psychometric properties of the measure in university students (Perz et al., 2020).

The aim of this study was to validate the FCV-19S in South Africa, examining the following psychometric properties of the measure in a sample of black African university students: (1) dimensionality, (2) discriminant validity and (3) reliability.

## Methods

### Participants

A convenience sample of 433 black African university students (Female = 58%, M_age_ = 23.51, standard deviation [SD] = 4.18) was used for the study. The sample was predominantly constituted by undergraduate students (88%) across the faculty of Humanities, whilst 76% of the participants resided in a rural area (see Table 1).

**TABLE 1:** Sociodemographic characteristics (*N* = 433).

Variable	*n*	%
**Gender**		
Female	254	58.7
Male	179	41.3
**Community**		
Urban	105	24.2
Rural	328	78.8
**Ethnic group**		
Black African	433	100
**Age groups**		
18 years	11	2.5
19–20 years	60	13.9
21–23 years	191	44.1
24–26 years	111	25.6
> 26 years	60	13.9
**Study year**		
1st year	55	12.7
2nd year	51	11.8
3rd year	129	29.8
4th year	133	30.7
Masters	52	12.0
Missing values	13	3.0

### Design

Data were collected online, within a cross-sectional design.

### Instruments

#### Fear of COVID-19 Scale

The seven-item FCV-19S (Ahorsu et al., 2020) examined COVID-specific anxiety on a Likert scale (i.e. 1 [strongly disagree] to 5 [strongly agree]). Items of the FCV-19S include: Item 1: ‘… most afraid of COVID-19’ and item 4: ‘… afraid of losing my life because of COVID-19’. Participants could achieve a score that ranges from 7 to 35. The measure achieved an internal consistency reliability of α > 0.80 in previous studies (e.g. Elemo et al., 2020; Perz et al., 2020; Soraci et al., 2020). In this study reliability was estimated at α = 0.88.

#### Jackson-5 Fear Scale

The J-5FS, a seven-item fear subscale of the Jackson-5 scales (Jackson, 2009), was used to measure ordinary fear. Its response scale is anchored from 1 [completely disagree] to 7 [completely agree], denoting that a high score is equivalent to a high fear report. Two items are reverse scored. Jackson (2009) reported a reliability estimate of α = 0.69 for the measure, whilst we found a modest α = 0.50 in the present study.

### Procedure

All participants consented to participation in the study before completing the questionnaire. Participants were recruited online using class registers. The registers were simply used to direct the communication to the potential respondents because they contained student-number formulated e-mail addresses (i.e. a university-generated e-mail address that only contains a student number and not the student’s name). They were directed to the website where the study questionnaire, designed using Google Forms, was posted. The survey was only in English and required between 20 and 30 min to complete. The total sample consists of two data sets (i.e. *n* = 202 and *n* = 231) that were collected around the same time, although data set 1 does not have all the measures covered in data set 2. Data were collected by two different research groups using the same data collection media, during the same period and utilising the same student population.

### Data analysis

#### Factor structure

Two data sets were used to conduct the main analyses. A combined data set (*n* = 433, combining data set 1 and 2) was used to examine the factor structure of the FCV-19S using confirmatory factor analysis (CFA). The CFA analyses (of the one-factor model consistently found in the literature) were conducted with the weighted least squares mean and variance adjusted (WLSMV) estimator for ordinal data in Mplus 7.4 (Muthén & Muthén, 2017). The model fit was evaluated with the comparative fit index (CFI), the Tucker–Lewis index (TLI), standard root mean residual (SRMR) and the root mean square error of approximation (RMSEA) (i.e. TLI and CFI ≥ 0.95 [adequate at 0.92 to 0.94] and RMSEA < 0.08) (Makhubela & Mashegoane, 2019). Two alternative models were also tested. The two-factor model consisted of the *fear thoughts* (items 1, 2, 4, 5) and the *physical response* factor (items 3, 6, 7). The bi-factor model incorporated a general fear factor and two orthogonal factors (i.e. fear thoughts and physical response).

#### Discriminant validity

Following CFA, only data set 2 (*n* = 231) was used to evaluate the discriminant validity of the measure using exploratory factor analysis (EFA). The dimensionality of the FCV-19S and J-5FS items under EFA was estimated with maximum likelihood using principal axis factoring (PAF) with varimax rotation. Factor selection was performed using parallel analysis (PA).

#### Reliability

Finally, based on results of the first analysis, the internal consistency, Omega and greatest lower bound (GLB) reliability estimates of the unidimensional scale of the FCV-19S were examined.

### Ethical considerations

Ethical compliance was approved by the Turfloop Research Ethics Committee of the University of Limpopo, reference number: TREC/375/2020: IR.

## Results

### Item analysis

Item mean, standard deviations and normality of the FCV-19S were examined. The univariate skewness and kurtosis for each of all the seven items of the scale are within the normal range of –1.5 to 1.5 (see Table 2).

**TABLE 2:** Normality, mean and standard deviation of the FCV-19S items.

Item	Skewness	Kurtosis	Mean	SD
FCV-19S 1	−0.56 (0.12)	−0.94 (0.23)	3.53	1.36
FCV-19S 2	−0.12 (0.12)	−1.27 (0.23)	3.12	1.36
FCV-19S 3	−1.04 (0.12)	−0.20 (0.23)	2.05	1.17
FCV-19S 4	−0.75 (0.12)	−0.81 (0.23)	3.71	1.41
FCV-19S 5	−0.17 (0.12)	−1.37 (0.23)	3.15	1.46
FCV-19S 6	0.97 (0.12)	0.07 (0.23)	2.02	1.13
FCV-19S 7	0.49 (0.12)	−1.00 (0.23)	2.46	1.34

Note: FCV-19S, Fear of COVID-19 Scale; SD, standard deviation.

### Confirmatory factor analysis

**FIGURE 1:**
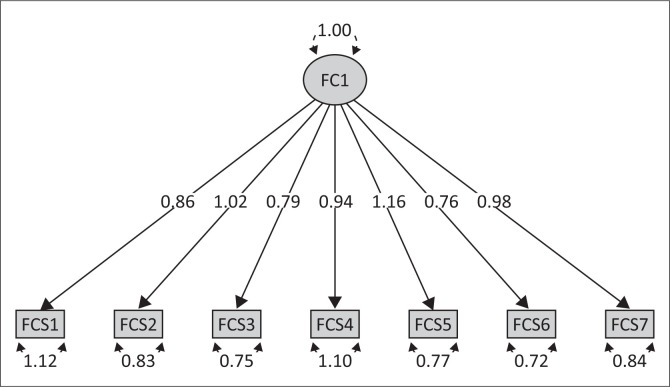
Fear of COVID-19 unidimensional structure.

Results of CFA conducted on the FCV-19S revealed a well-fitting model (see Figure 1) to the data (*X*^2^ = 51.637, degrees of freedom [df] = 14, *p* < 0.001, TLI = 0.975, CFI = 0.983, SRMR = 0.070, RMSEA = 0.079, with a 90% CI [0.057–0.102]) and all parameters were viable. Two alternative models were also tested: two-factor model (*X*^2^ = 55.687, df = 13, *p* < 0.001, TLI = 0.817, CFI = 0.887, SRMR = 0.041, RMSEA = 0.087, with a 90% CI [0.064–0.111]) and bi-factor model (*X*^2^ = 22.793, df = 7, *p* < 0.005, TLI = 0.966, CFI = 0.989, SRMR = 0.033, RMSEA = 0.072, with a 90% CI [0.041–0.106]). Whilst the bi-factor model appears on the basis of fit indices to have a good fit, the model is however a poor model because of the fact that not all model parameters were viable (i.e. items 2 and 4 load poorly on the first factor). As such the overall results did not support the two alternative models.

### Discriminant validity

The FCV-19S and the J-5FS were loaded and factor analysed together to establish discriminant validity. The Kaiser–Meyer–Olkin (KMO) measure was 0.86 showing that the sample size used in the study was adequate for EFA (Field, 2009), whilst the Bartlett test of sphericity was less than the critical level of significance (*X*^2^ = 1410.98, *df* = 21, *p* < 0.001) indicating that the data were suitable for EFA. Principal axis factoring produced a two-factor solution. Table 3 shows the factor loadings (> 0.30) and there were no cross-loading items. Two items of the J-5FS (i.e. J-5FS 1 and 4) did not load on either of the two factors. In sum, the EFA results suggest that there is discriminant validity between the FCV-19S and the fear items of the J-5FS.

**TABLE 3:** Factor matrix of the Fear of COVID-19 Scale and the Jackson-5 Fear Scale.

Item descriptor	Principal factors	
	Factor 1	Factor 2
FCV-19S 1	0.68	-
FCV-19S 2	0.69	-
FCV-19S 3	0.63	-
FCV-19S 4	0.69	-
FCV-19S 5	0.75	-
FCV-19S 6	0.63	-
FCV-19S 7	0.68	-
J-5FS 2	-	0.38
J-5FS 3	-	0.40
J-5FS 5	-	0.61
J-5FS 6	-	0.60
J-5FS 7	-	0.65

FCV-19S, Fear of COVID-19 Scale; J-5FS, Jackson-5 Fear Scale.

### Reliability

The reliability estimates of the FCV-19S were acceptable (α = 0.87, ω = 0.88 and GLB = 0.90).

## Discussion

This study set out to evaluate the psychometric properties of the FCV-19S in the South African context, using a predominantly black African student population in the Limpopo province. The CFA results lend support to a unidimensional factor structure of the scale (Ahorsu et al., 2020; Alyami et al., 2020; Elemo et al., 2020; Mailliez et al., 2020; Nguyen et al., 2020; Pang et al., 2020; Reznik et al., 2020; Sakib et al., 2020; Satici et al., 2020; Soraci et al., 2020; Tsipropoulou et al., 2020; Winter et al., 2020). Discriminant validity was established using a sample size that can be considered to be large enough to provide stable factors (KMO measure ≥ 0.80; Field, 2009). Items of the FCV-19S and the J-5FS loaded separately, with all seven FCV-19S items loading on their own factor. The results demonstrate that the fear measured by the FCV-19S is unique to COVID-19 (Perz et al., 2020) and therefore worthy of being studied as a stand-alone construct.

In spite of the FCV-19S being unidimensional, there is a pattern of response to the items where endorsements of items 3, 6 and 7 are comparatively low when contrasted with the scores of the remaining items. The pattern was observed in mean scores reported by studies such as Elemo et al. (2020), Giordani et al. (2021), Pang et al. (2020), Perz et al. (2020), Sakib et al. (2020), Satici et al. (2020), Soraci et al. (2020) and Winter et al. (2020). A closer inspection of the items shows that they refer to physiological reactions because of COVID-19 fear (Alyami et al., 2020). The pattern of response most likely explains why some studies (Bitan et al., 2020; Chi et al., 2020; Masuyama et al., 2020) obtained a second factor in their factor analytic studies, which comprises the three low-scoring items.

The reliability estimate of the FCV-19S is comparable with those obtained in many other studies from different geographic contexts (cf. Elemo et al., 2020). The Omega obtained in this study (ω = 0.88) is the same as that found by Elemo et al. (2020) and the GLB is nearly the same as that obtained by Pang et al. (2020). The reliability of the scale implies that the scores can be trusted because they can be reproduced whenever the measure is administered. Reproducibility improves confidence in making clinical and other decisions of intervention. Decisions can be based on the results obtained with the FCV-19S. For instance, results of the FCV-19S can help in the pitching of messages related to COVID-19 because it is well-known that extreme fear and subsequent panic during a pandemic tend to minimise the reception of communications related to the illness (Ng & Kemp, 2020). The reason for that is as follows: beyond the fear of potential infection, fear of the unknown related to COVID-19 has been shown to give rise to clinical anxiety symptoms, also affecting the mental health of healthy people (Shigemura, Ursano, Morganstein, Kurosawa, & Benedek, 2020). Valid COVID-19-related mental health screeners are necessary to assist with the early identification of people at risk of pandemic-related psychological distress, to enable preventative and supportive interventions. Whilst there are a number of mental health screeners associated with COVID-19, the FCV-19S has advantage over many of them because of its shortness, making it more appropriate for resource and time constrained student counselling contexts. This benefit is also true for research purposes.

The limitation of the study is that the sample was not randomly drawn and therefore may not likely be completely representative of the targeted study population. There are more students domiciled in a rural area and they were over-represented in this sample. The study has to be replicated in a different research site and with non-student samples to confirm the results. Additional psychometric properties that could offer more validity evidence for the FCV-19S, such as predictive validity, convergent validity and measurement invariance, were not assessed.

## Conclusion

This study established that the FCV-19S can be used as a unidimensional measure of COVID-19-related fear amongst university students in South Africa. It is also reliable. The FCV-19S has also been shown to be a construct distinct from ordinary fear. Its scores will assist with messaging pertaining to COVID-19 prevention.
